# Creep Behavior Characterization of Nickel-Based Single-Crystal Superalloy DD6 Thin-Walled Specimens Based on a 3D-DIC Method

**DOI:** 10.3390/ma16083137

**Published:** 2023-04-16

**Authors:** Yue Zhang, Jiangkun Hu, Lixia Kang, Yuhuai He, Wei Xu

**Affiliations:** 1 Beijing Key Laboratory of Aeronautical Materials Testing and Evaluation, AECC Beijing Institute of Aeronautical Materials, Beijing 100095, China; 2 School of Measuring and Optical Engineering, Nanchang Hangkong University, Nanchang 330063, China; 3 Unit 63615 of PLA, Beijing 100071, China

**Keywords:** creep, single-crystal superalloy, thickness debit effect, digital image correlation

## Abstract

The thickness debit effect of creep behavior has been a focal point of nickel-based single-crystal superalloy research, and there is a need for an advanced creep deformation measurement method. This study developed a novel high-temperature creep test system based on a single-camera stereo digital image correlation (DIC) method with four plane mirrors to conduct creep tests on thin-walled specimens of a nickel-based single-crystal alloy, DD6, with thicknesses of 0.6 mm and 1.2 mm under experimental conditions of 980 °C/250 MPa. The reliability of the single-camera stereo DIC method in measuring long-term deformation at a high temperature was experimentally verified. The experimental results show that the creep life of the thinner specimen was significantly shorter. It was found the lack of coordination in the creep deformation process of the edge and middle section of the thin-walled specimens may be an important factor in the thickness debit effect according to the full-field strain contour. By comparing the local strain curve at the rupture point with the average creep strain curve, it was found that the creep rate at the rupture point was less affected by the specimen thickness during the secondary creep stage, while the average creep rate in the working section significantly increased as the wall thickness decreased. The thicker specimen usually had a higher average rupture strain and higher damage tolerance, which prolonged the rupture time.

## 1. Introduction

Nickel-based single-crystal superalloys, which have an excellent high-temperature mechanical performance, serve as advanced materials for the manufacturing of advanced aerospace engines and gas turbine blades. In the aerospace engine, the turbine blades withstand centrifugal loads caused by wheel rotation in high-temperature environments, leading to creep rupture problems. Therefore, the study of creep properties under high-temperature conditions is of great reference significance for the material selection and mechanical design of turbine blades [1,2,3,4].

With the improvement of aerospace engine performance, thin-walled structures of a thickness of less than 1 mm with air-cooling function have been widely used in high-pressure turbine blades. Previous research has shown that structure thickness has a significant influence on the creep performance of single-crystal superalloys. Doner et al. [5] first discovered that the creep performance of the nickel-based single-crystal alloy CMSX-3 has a significant thickness dependence, and the sustained rupture time of thin-walled specimens with a thickness of less than 1 mm is more than 30% lower than that of standard specimens. The phenomenon of performance degradation, caused by a decrease in thickness, is known as the thickness debit effect. The thin-walled region of turbine blades is often a weak link in their performance, and their mechanical properties determine the overall performance and service life of the component. Therefore, it is necessary to study the influences of thin-walled structures on the mechanical properties of superalloys. Srivastava et al. [6] investigated the creep behavior of PWA1484 single-crystal nickel-based alloy thin-walled specimens under experimental conditions of 760 °C/758 MPa and 982 °C/248 MPa. The results showed that the thickness of the specimens had little effect on the steady-state creep rate at 760 °C/758 MPa, and the thickness debit effect was mainly caused by cleavage cracks induced by deformation in thin specimens. In contrast, at 982 °C/248 MPa, the steady-state creep rate decreased with the thickness of the specimen. Hu et al. [7] conducted creep rupture experiments on thin-walled specimens of nickel-based single-crystal superalloys of different thicknesses under conditions of 980 °C/250 MPa. The results showed that cracks and creep voids were the causes of the thickness debit effect: creep crack propagation played a dominant role in the occurrence of the thickness debit effect for thin specimens, while creep damage played a dominant role for thick specimens.

In creep behavior research on single-crystal superalloys, creep axial strain is usually measured using mechanical or electronic extensometers, but the tertiary creep stage typically manifests as non-uniform plastic deformation. In this case, the full strain field of creep experiments cannot be obtained through high-temperature extensometers. Due to the limitations of contact measurement methods in some application areas, non-contact optical methods based on digital image correlation (DIC) technology have been introduced into experimental mechanics and have gradually gained widespread use in the field of full-field strain measurement. The digital image correlation method was initially proposed by Peters [8], from the University of South Carolina in the United States, and Yamaguchi [9], from Japan, and has now developed into a relatively mature and widely applied non-contact testing method. Zheng et al. [10] measured the influence of cyclic thermal loading on the fatigue creep behavior of pure copper specimens using DIC technology and found that under certain loads, an increase in the temperature rise rate caused the pure copper specimen to enter the fast creep stage earlier. Shang and Dong [11,12] utilized the full-field capability of UV-DIC technology to characterize the creep behavior of nickel-based superalloys and demonstrated the superiority of the DIC method. However, due to off-plane displacement and lens distortion, the measurement system error of the 2D-DIC method is difficult to overcome. Therefore, 3D-DIC technology, which can not only achieve the deformation measurement of curved surfaces but also overcome some system errors such as off-plane displacement and lens distortion, thereby improving the measurement accuracy, has been developed [13,14,15,16]. In recent years, the single-camera 3D-DIC method has been developed to measure three-dimensional deformation through a single camera and a spectroscope system instead of two cameras. Its greatest advantages are that the acquisition of 3D images does not require synchronization between two cameras and that insufficient testing space can be expanded to a large extent when testing in a furnace for high-temperature tests. 

In order to study the thickness debit effect, it is necessary to obtain deformation data. However, traditional strain gauge methods can only obtain average strains in a single direction and cannot provide information about local area deformation [17,18]. Therefore, single-camera 3D-DIC technology has been shown to have advantages such as a high testing accuracy, cost-effectiveness, and small space occupation and can be used for experiments at the same working distance without the need for calibration before each experiment, compared with traditional methods. In the study of the thickness debit effect, single-camera 3D-DIC technology can provide deformation characteristics in the depth direction of the measurement object, which better explains the thickness debit effect. Yu et al. [19], through experiments, verified that the displacement measurement accuracy of the single-camera 3D-DIC system is high, and it can measure the full-field deformation of non-directly heated surfaces (rear surfaces) of stainless steel and alumina ceramic specimens at different temperatures. Moreover, the results are consistent with the reference values in aerospace material manuals, demonstrating the reliability of this technology.

This paper investigates the thickness dependency of creep properties of a second-generation nickel-based superalloy, DD6, using single-camera 3D-DIC technology. Single-axis creep experiments were conducted on DD6 thin-walled specimens with different thicknesses and [001] orientation at 980 °C/250 MPa. By analyzing the calculated creep curves and three-dimensional full-field deformation contour maps, the creep performance characteristics of the nickel-based single crystal superalloys of different thicknesses were explored under the experimental conditions of 980 °C/250 MPa.

## 2. Methods

The measurement of the 3D deformation and displacement of the measured specimen was achieved using the 3D-DIC method, which is based on the principle of binocular stereovision. The problem of serious errors caused by off-plane displacement during the measurement process with the 2D-DIC method was solved. The emergence of the single-camera 3D-DIC technology measurement system solves the disadvantages of the dual-camera system, such as its difficult calibration, complex equipment, inconvenience when carrying, and inability to be used in small test environments. Its implementation principle is to realize the binocular testing function of a single camera through auxiliary devices that change the light path, such as diffraction gratings, prisms [20,21], and four-plane mirrors [22]. Compared with the other two auxiliary devices, the four-plane mirror has the advantages of low cost, less distortion, and a higher spatial resolution.

**Figure 1:**
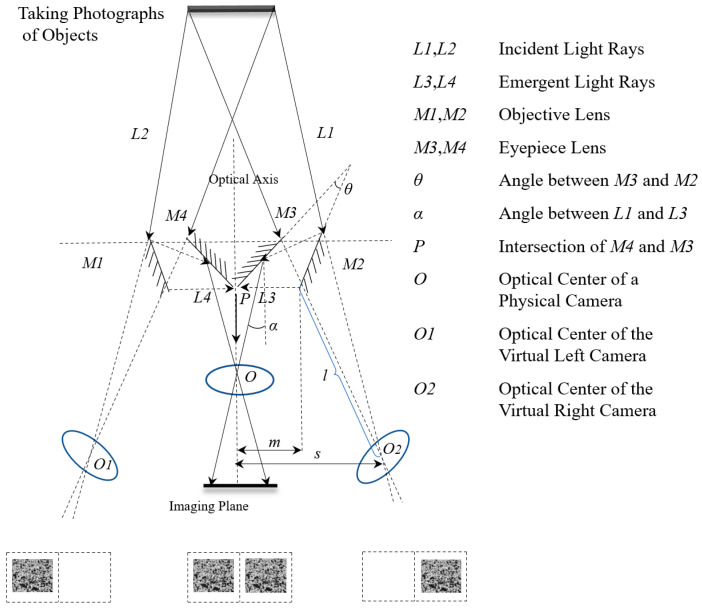
Structure drawing of DIC system with auxiliary device of four flat mirrors.

The experiment described in this paper employed a single-camera stereo-DIC testing system, as shown in Figure 1. The system utilized an auxiliary device consisting of four flat mirrors to convert a single camera into two virtual cameras. The left and right virtual cameras each covered half of the original camera’s field of view. Additionally, the system captured images of the specimen at different angles, eliminating the need for the complex synchronization calibration required by a dual-camera setup.

The single-camera stereo-DIC measurement system is designed to produce images that are symmetric with respect to the optical axis. Therefore, the right half of the optical path is analyzed, and the angle between the inner mirror and outer mirror on the right side is denoted as *θ*, while the angle between the outgoing ray and the incoming ray in the opposite direction is denoted as *α*. Based on the reflection principle of the optical path, the mathematical relationship expressed in Equation (1) can be obtained.

(1) α=2θ

The calculation formula for the distance *Ɩ* from the center of the virtual camera *O2* along the optical axis to *M2*, as well as the horizontal distances from the center of the virtual camera *O2* along the optical axis to the optical axis of the real camera *O*, can be obtained by combining Equation (1) with the relationship between the position *O* of the real camera and the symmetry of the optical axes of the virtual cameras *O1* and *O2* with respect to the plane mirrors *M2* and *M3*, as shown in Figure 1.

(2) l=m+n

(3) s=l×sin⁡α+m=(m+n)×sin⁡(2θ)+m

As shown in Figure 1, *m* represents the distance between point *P* (the intersection of the two inner mirrors) and *M2*, and *n* represents the distance between point *P* and *O*.

**Figure 2:**
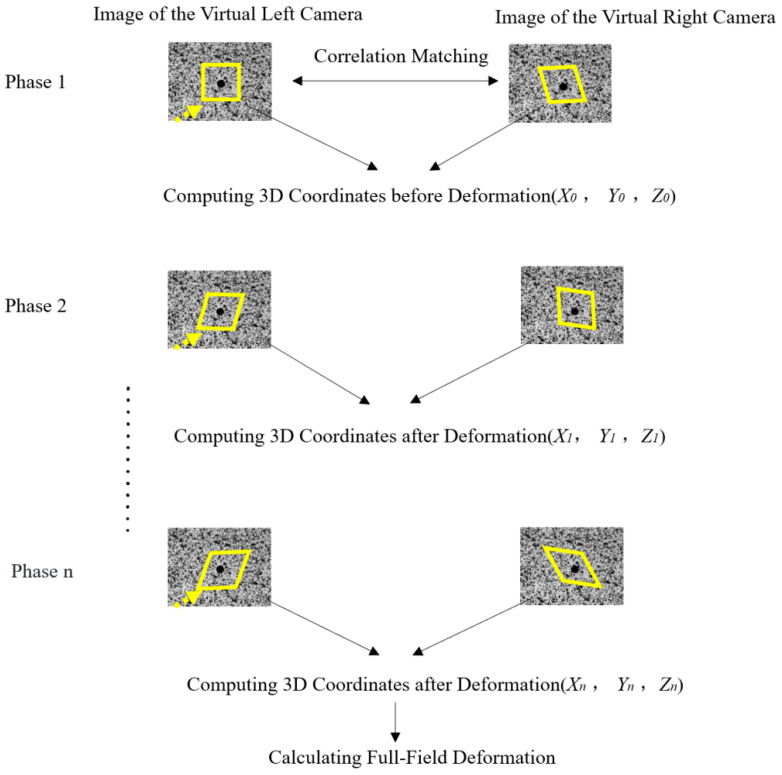
Flow chart of 3D-DIC method.

The flow chart of 3D-DIC method is shown in Figure 2. The calculation process of 3D-DIC for a single camera can be divided into four parts: (1) image acquisition—a pair of virtual cameras can be formed by combining the single camera with auxiliary equipment, resulting in a set of images captured from different directions; (2) image matching—appropriate reference sub-regions are selected based on the image captured by the left virtual camera, and a region-based stereo matching algorithm is used to search for corresponding sub-regions in the image captured by the right virtual camera; (3) 3D reconstruction—the three-dimensional coordinates of the sub-region can be obtained by solving a set of corresponding two-dimensional coordinates; and (4) 3D strain calculation—the full-field strain can be determined by computing the three-dimensional spatial coordinates of the test points before and after deformation.

In order to achieve image matching and evaluate speckle quality, in this paper, we employ an algorithm called zero-mean normalized sum of squared difference criterion (ZNSSD) to calculate the correlation between images at different time points. The expression for ZNSSD is shown in Equation (4).

(4) $$C_{\mathrm{ZNSSD}\;}=\sum_{i=-M}^{M}\;\sum_{j=-M}^{M}\;\left\{\frac{f\left(x_{i},y_{j}\right)-f_{m}}{\sqrt{\sum_{i=-M}^{M}\;\sum_{j=-M}^{M}\;{\left[f\left(x_{i},y_{j}\right)-f_{m}\right]}^{2}}}-\right.\left.\frac{g\left(x_{i}^{\prime },y_{j}^{\prime }\right)-g_{m}}{\sqrt{\sum_{i=-M}^{M}\;\sum_{j=-M}^{M}\;{\left[g\left(x_{i}^{\prime },y_{j}^{\prime }\right)-g_{m}\right]}^{2}}}\right\}$$

In this context, *i* represents the length and *j* represents the width of the image. $f\left(x_{i},y_{j}\right)$ represents the grayscale value of any point $\left(x_{i},y_{j}\right)$ in the reference image, while $g\left(x_{i}^{\prime },y_{j}^{\prime }\right)$ represents the grayscale value of any point $\left(x_{i}^{\prime },y_{j}^{\prime }\right)$ in the target image. Furthermore, *f*_m_ and *g*_m_ represent the average grayscale values of the reference and target images, respectively. 

## 3. Material and Experimental Procedure

### 3.1. Material 

The experiment was conducted using specimens cut from a nickel-based single-crystal superalloy DD6 ingot. The nominal chemical composition (wt%) of the alloy was as follows: Cr 4.3, Co 9, Mo 2, W 8, Ta 7.5, RE 2, Nb 0.5, Al 5.6, Hf 0.1, C 0.006, and Ni balance. The specimens were subjected to the standard heat treatment process (1290 °C × 1 h + 1300 °C × 1 h + 1318 °C × 4 h/AC + 1120 °C × 4 h/AC + 870 °C × 32 h/AC). The mechanical properties of the DD6 alloy of [001] orientation are shown in Table 1.

**Figure 3:**
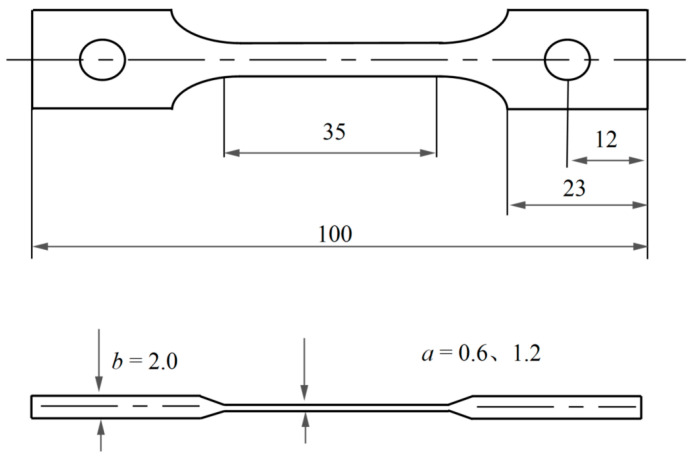
Geometry of the present thin-walled creep specimen.

Using wire-cutting technology, initial plate-shaped specimens were cut from the blank with the primary orientation of [001] and secondary orientation of [010]. The dimensions of the specimens varied, as shown in Figure 3. Finally, the surfaces of the specimens were polished with SiC sandpaper to remove machining marks, resulting in the final specimens for this experiment.

**Figure 4:**
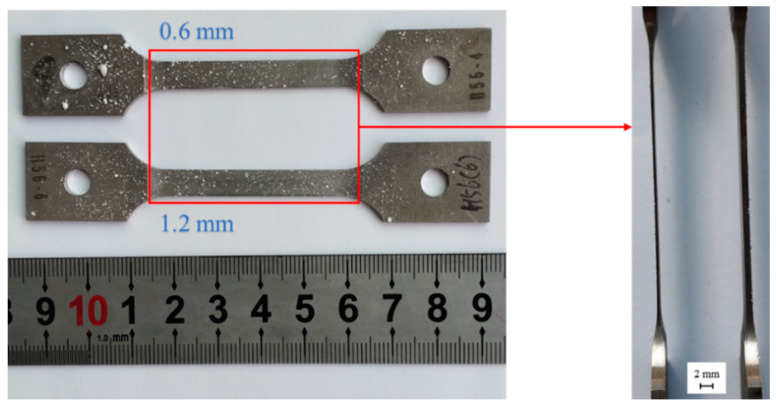
Photo of creep specimens with thickness of 0.6 mm and 1.2 mm after pretreatment.

The marking and recognition of the specimen surface in DIC methods necessitates the use of speckles, which should possess features such as high contrast and resistance to detachment at high temperatures. In this study, inorganic high-temperature paint was prepared by uniformly mixing aluminum oxide powder and inorganic solvent in a 3:1 ratio. Firstly, a layer of randomly distributed, uniform, and high-contrast speckles was formed on the specimen surface by dripping. The prepared speckle specimens were allowed to air-dry naturally for 2 h at room temperature before being placed in a high-temperature chamber at 93 °C and heated for 3 h. After cooling, loosely attached speckles were blown off using an ear syringe to complete the production of high-temperature speckles, and the final preprocessed specimens are shown in Figure 4.

### 3.2. Experimental Procedure 

The experimental system consists of a creep loading device and a single-camera 3D-DIC testing system. The creep loading device includes a furnace and a persistent creep testing machine (maximum test loading of 10 kN). The high-temperature muffle furnace has a high temperature control accuracy. Factors such as the thickness, surface curvature, and refractive index of the glass in the observation window may cause calculation errors. Non-uniform refraction of the glass during creep can cause differences in the left and right visual fields, leading to local distortion in the 3D imaging of the surface, which affects the calculation results. Therefore, high-quality quartz glass is used on the observation window to reduce the impact of glass refraction on the stereoscopic matching of 3D images. At the same time, the observation window is equipped with a simple shading device to adjust the size of the observation window, which ensures that the experimental device is not overheated and sufficient light can enter so as to obtain high-quality images.

**Figure 5:**
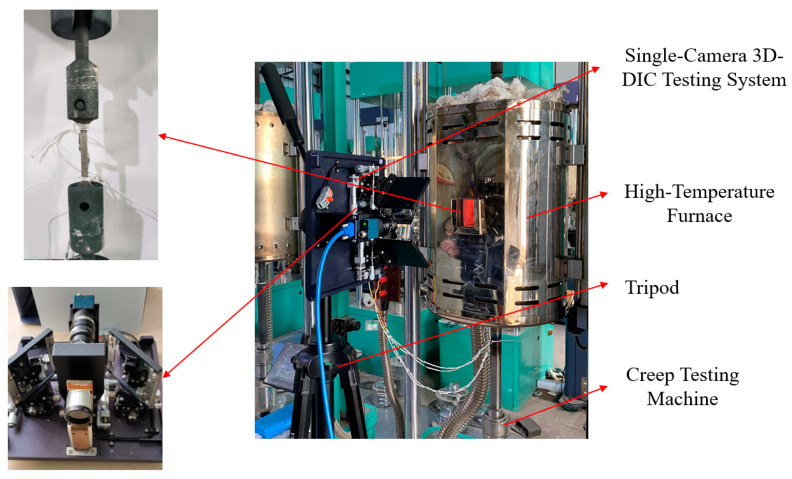
Creep test system based on 3D-DIC method.

The single-camera 3D-DIC testing system used in this study consists of an industrial camera (model: MER-1070-14U3M/C, resolution: 3840(H) × 2748(V)), a blue light source controller, a narrowband optical filter (passband: 430–470 nm), and a computer for data recording. The calibration board is a 7 × 7 chessboard grid calibration board, with each grid having a side length of 4 mm. After the initial calibration, no further calibration is needed for subsequent experiments. The working distance of the single-camera 3D-DIC testing system is 550 mm, and the field of view size is 165 mm × 105 mm. During the high-temperature experiment, the system provides adjustable blue illumination through the light source controller and suppresses the influence of thermal radiation on the speckle image using a narrowband filter. This filter can effectively filter out infrared radiation in the thermal radiation. The final experimental system is shown in Figure 5.

During the creep deformation testing in this study, we used a data-averaging method to eliminate the influence of test noise on the measurement results. A total of 30 images were obtained within 5 min, and the average value of these 30 data points was used as the final measurement value. The fluctuation in these test points is affected by the level of noise, and their variance reflects the precision of the measurement [14]. 

### 3.3. Accuracy Verification

**Figure 6:**
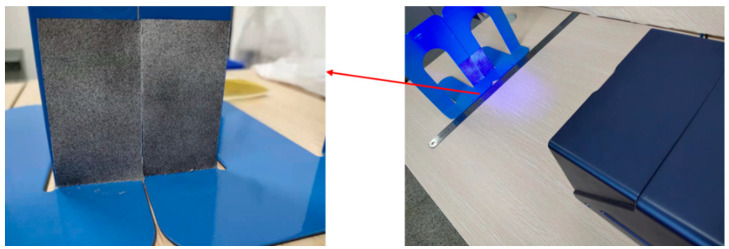
Accuracy verification of single-camera 3D-DIC test system in this study.

Prior to the formal experiment, the single-camera 3D-DIC testing system needs to be verified. The test involves placing two movable square brackets closely together and spraying the central portion of the brackets with the prepared speckle, as shown in Figure 6. The left and right brackets are moved in parallel, and the distance moved is measured using a ruler. The measurement results obtained using the single-camera 3D-DIC testing system are then compared with those obtained using the ruler. The experimental results are shown in Table 2, which indicates that the relative error of the measurement accuracy of the single-camera 3D-DIC testing system is within 5% (the relative error refers to the ratio between the absolute error and the reference value of the ruler). 

After comparison with the test results of other types of experimental equipment [14,15,16,17], the single-camera 3D-DIC testing system used in this experiment showed a higher accuracy and precision in displacement measurement at room temperature than the ordinary 2D-DIC equipment. Its accuracy is similar to that of other stereo-DIC instruments and 2D-DIC video extensometers based on telecentric lenses. In addition, its precision is slightly higher than that of single-camera 3D-DIC methods employing other spectroscope systems. The possible reason for this is that the integrated four-mirror optical splitting system has higher vibration stability. 

### 3.4. Speckle Quality Assessment 

**Figure 7:**
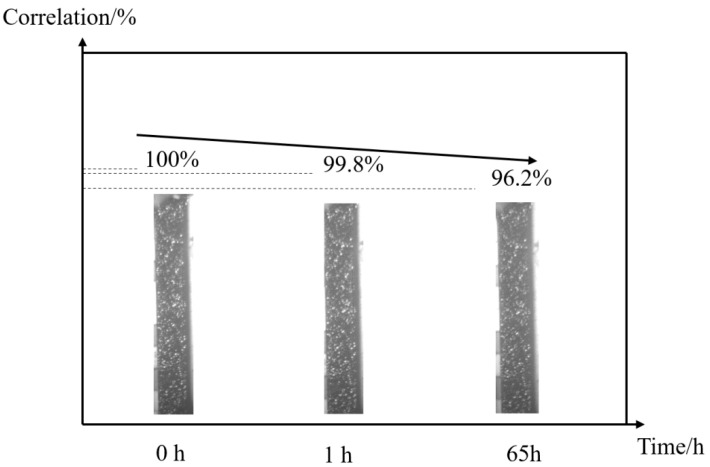
Speckle evolution assessed by correlation calculation during the creep process.

Before the experiment, the parameters of the four-mirror optical splitting system were set, and the camera’s shooting angle and distance were adjusted to obtain clear images. The camera’s sampling frequency was 1 frame per minute, and the collected images were transmitted to a computer for storage. After the sampling began, the specimen was loaded, and the experimental stress was maintained at 250 MPa. Taking the images collected at 1 h and 65 h as examples, as shown in Figure 7, it can be observed that there is no obvious spalling of the speckle on the specimen surface, and the contrast is still acceptable. The correlation calculation was performed using the zero-mean normalized sum of squared difference (ZNSSD) function with strong anti-interference and good single-peak characteristics, as shown in Equation (4). From the calculation results shown in Figure 7, it can be seen that the similarity between the speckle specimen images at the initial time and 65 h later does not differ by more than 5% (including the similarity difference caused by specimen deformation), which indicates that the high-temperature speckle produced in this experiment did not fall off under high-temperature conditions and still had good identification characteristics after long-term experimentation. Additionally, the combination of blue light and filters used in the single-camera 3D-DIC system can effectively suppress the influence of thermal radiation on imaging. 

## 4. Results and Discussion

### 4.1. Creep Behavior of the Thin-Walled Specimens

**Figure 8:**
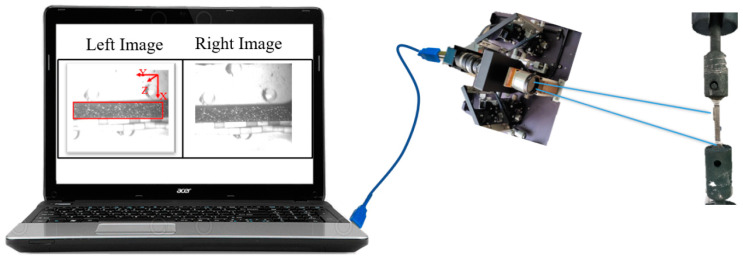
Schematic diagram of the specimen surface for observation and calculation.

**Figure 9:**
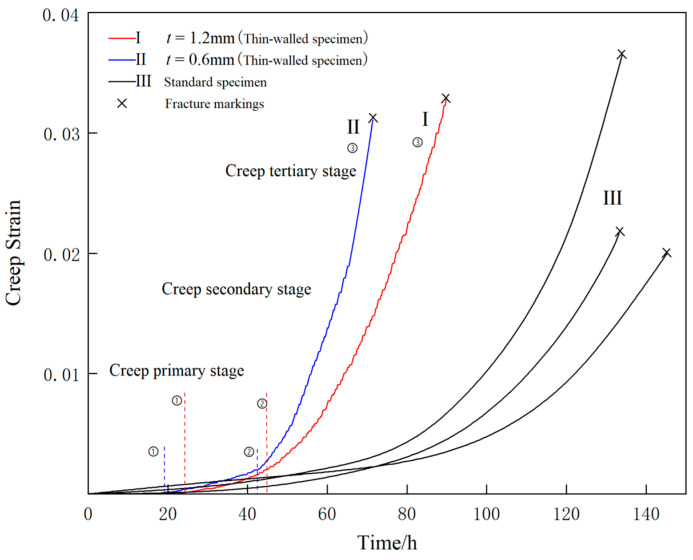
The average creep strain vs. time curves of the thin-walled specimens and standard specimens at 980 °C/250 MPa.

During the experiment, images were collected to record the creep deformation information of the specimen. The collected images were subjected to preliminary image processing, and the invalid regions were removed to improve the calculation efficiency. Then, using the left part of the image as the reference image, the region was calculated as shown in the boxed part of Figure 8. The region size is 250 pixels × 50 pixels, with a subregion size of 25 pixels × 25 pixels and a step size of 2 pixels during calculation. Finally, the creep strain versus time curves for specimens of different thicknesses were obtained under the same conditions (980 °C, 250 MPa), as shown in Figure 9. Due to the gap between the mechanical components of the creep testing machine, the true value of the initial strain (ε_0_) was seriously affected during loading. To ensure data reliability, the initial strain was not considered for the creep curves presented in this paper. 

Figure 9 shows the creep curves in the y-axis direction for specimens with thicknesses of 1.2 mm and 0.6 mm under experimental conditions of 980 °C/250 MPa. Based on the creep rate as a criterion used to divide different creep stages, the different creep stages are marked as ①, ②, ③, etc. As shown in Figure 9, the creep rate gradually decreased during the first stage, remained almost constant during the second stage, and gradually increased during the third stage.

As shown in Figure 9, comparing the strain curves in the stretching direction between specimens with thicknesses of 0.6 mm and 1.2 mm, it can be seen that the performance of the two specimens was not significantly different during the first and second creep stages, but the second creep stage of the thinner specimen was shorter than that of the thicker specimen. After entering the tertiary creep stage, the creep rate gradually increased, and significant differences were observed between the two thicknesses. The creep rupture time for the 0.6 mm thick specimen was approximately 20% shorter than that of the 1.2 mm thick specimen, showing a clear correlation between the specimen thickness and creep rupture life. Comparing the creep curves of thin-walled specimens and cylindrical specimens, it can be seen that the creep rate of the thin-walled specimen was significantly higher than that of the cylindrical specimen during the tertiary creep stage, and its creep life was significantly lower than that of the cylindrical specimen. 

**Figure 10:**
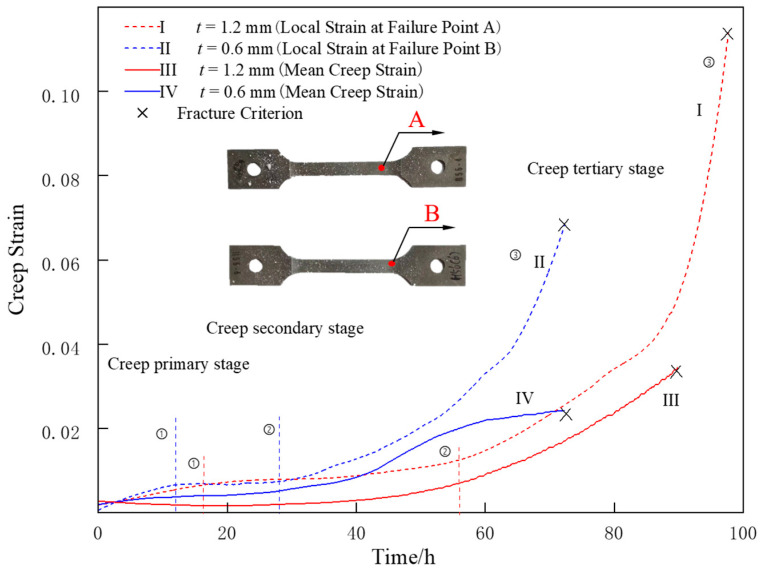
The curves of local strain vs. time and average strain vs. time of the thin-walled specimens of two thicknesses tested at 980 °C/250 MPa.

Nickel-based single-crystal superalloys have nickel-based solid solution γ phase matrix, where the γ′ phase (i.e., Ni3(Al, Ti, Ta)) is uniformly distributed, in concordance with [24,25]. Reed [26] studied the creep behavior of nickel-based single-crystal superalloys at different temperatures and stress levels. In the intermediate temperature range (800 °C~950 °C), the first creep stage was omitted, and the creep strain rate increased monotonically with the accumulation of creep deformation until rupture. With increasing temperature, dislocation climb and cross slip developed extensively at the γ/γ′ interface, leading to creep void damage, resulting in a monotonic increase in the creep rate. However, there has been no study on local damage behavior before creep rupture. Therefore, in this paper, the ability of DIC technology to measure full-field deformation was exploited to calculate the local strain and average creep strain curves at the rupture point for specimens of different thicknesses, as shown in Figure 10. The creep rupture point of 1.2mm and 0.6mm thick specimens are respectively defined as A and B.

**Figure 11:**
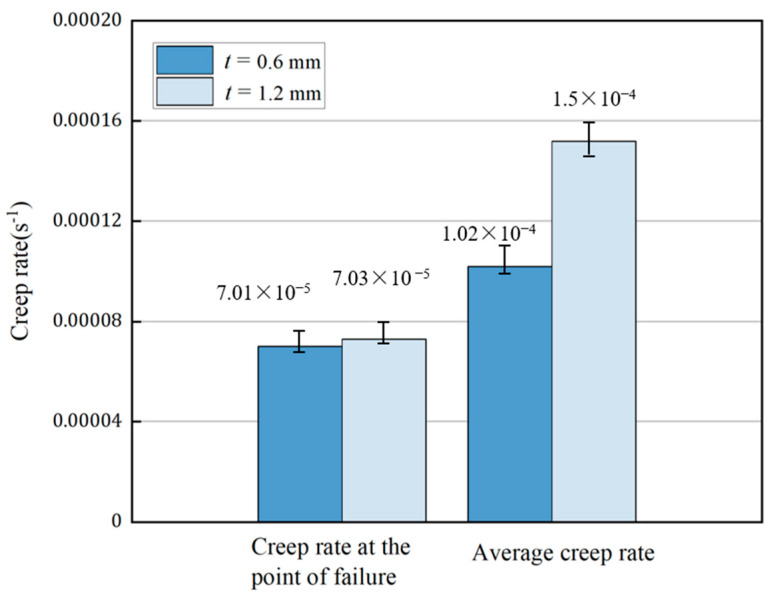
Comparison of the creep rate at the rupture point and the average creep rate during the second creep stage of the specimens of two thicknesses.

The results of calculating the creep rate at the rupture point and the average creep rate in the working section during second creep stage are shown in Figure 11. Comparing the creep rate curve at the rupture point and the average creep rate curve in the working section, it is found that during the second creep stage, the creep rate at the rupture point is less affected by the wall thickness, while the average creep rate in the working section increases significantly with the decreasing wall thickness. This suggests that the criterion of the tertiary creep stage can be presented as the maximum local creep rate. When the local creep rate of the most dangerous region reaches the critical value, the material enters the tertiary creep stage with the increasing creep rate. During the tertiary creep stage, both the creep rate at the rupture point and the average creep rate in the working section significantly increase with the decreasing wall thickness, but the increase in the creep rate at the rupture point is significantly higher than the average increase. 

### 4.2. Full Field Deformation Characterization

Srivastava et al. showed [6] that the thickness debit effect of the creep performance of single-crystal superalloys at 980 °C is mainly derived from two aspects. The first is that as the specimen becomes thinner, the critical crack length for rapid rupture decreases, resulting in a decrease in the creep rupture strain. The second is the decrease in the effective carrying capacity due to oxidation effects. This paper uses the advantages of DIC full-field deformation measurement to quantitatively analyze the source of the thickness debit effect. From the creep curves of the specimens of different thicknesses at their respective rupture points shown in Figure 10, it can be seen that the difference in the creep rate between the specimens of two thicknesses was not significant at the beginning of the test, but with the accumulation of creep deformation, the rupture strain at the danger point of the 1.2 mm thick specimen was approximately 50% higher than that of the 0.6 mm thick specimen, and the difference in the deformation capacity at the rupture point was more significant than the difference in the average rupture strain. Therefore, this paper hypothesizes that the creep rupture stage plays a more significant role in the thickness debit effect and thus concludes that among the factors that cause the thickness debit effect, the reduction in the critical crack length has a more significant impact.

**Figure 12:**
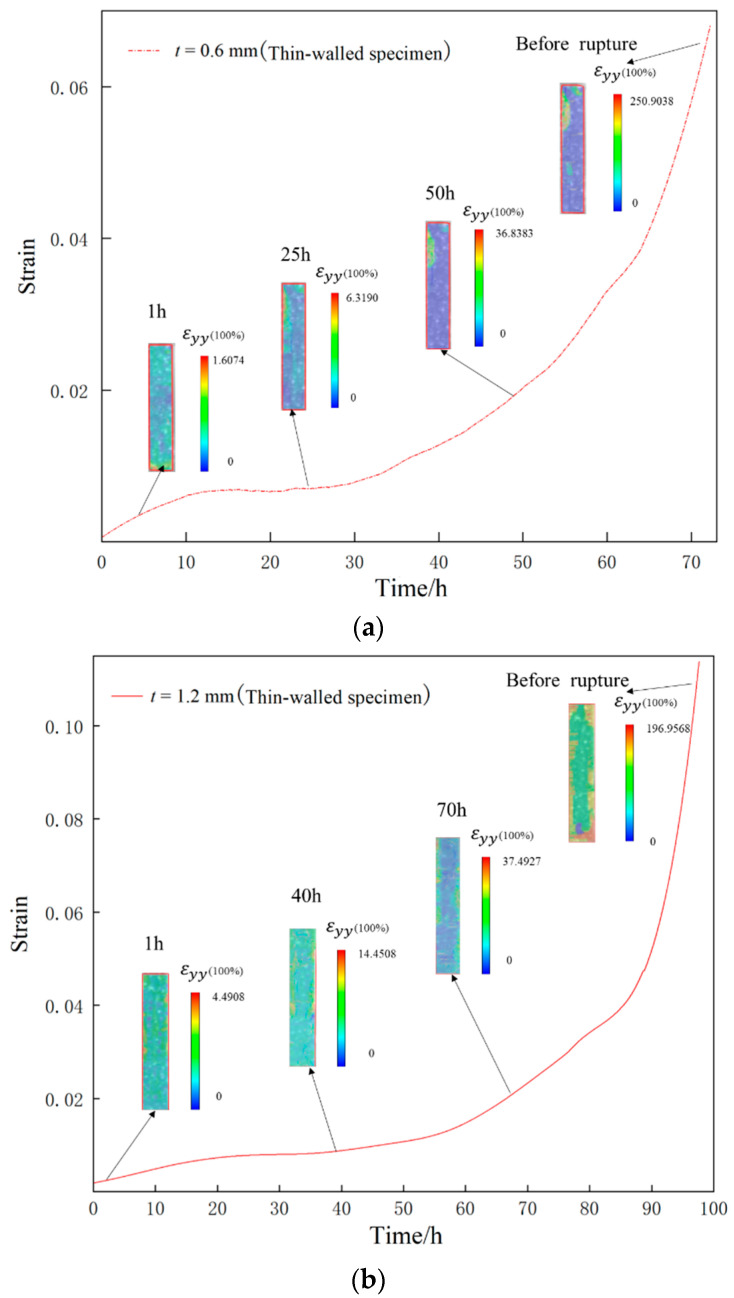
Full-field creep strain *ε*_yy_ contour maps of the thin-walled specimens tested at 980 °C/250 MPa: (**a**) 0.6 mm thick specimen, (**b**) 1.2 mm thick specimen.

**Figure 13:**
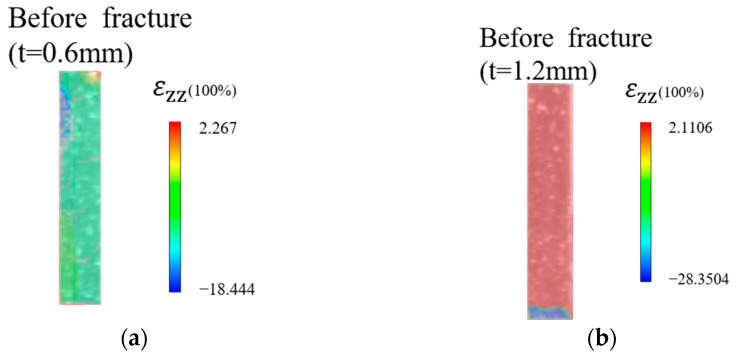
Full-field strain *ε*_zz_ contour maps of the thin-walled specimens before rupture tested at 980 °C/250 MPa: (**a**) 0.6 mm thick specimen, (**b**) 1.2 mm thick specimen.

The full-field strain (*ε*_yy_) contour maps showing the evolution of the specimens of two different thicknesses during the loading history were obtained by calculation, as shown in Figure 12. From the figure, it can be seen that both the thin-walled specimens have greater creep strain in the edge region, leading to stress concentration and finial rupture. The 0.6 mm thick specimen deformed relatively uniformly before 25 h, without obvious stress concentration. After 25 h, the local strain began to increase, and obvious stress concentration appeared on the edge of the specimen, which persisted until rupture, while the 1.2 mm thick specimen did not show obvious stress concentration until 70 h later. Comparing the full-field deformation contour maps of the specimens of two thicknesses before rupture, it can be seen that there is obvious stress concentration at the edge of the specimen. From the post-rupture specimens in Figure 13, it can be seen that the growth of creep voids mainly occurred at both ends of the specimen, thereby confirming the phenomenon of stress concentration at the edge of the working section on the contour map. This phenomenon may be caused by the water-quenching effect, because water quenching can cause uneven temperature gradients between the edge and interior of the specimen, resulting in probable stress concentration at the edge. In addition, the specimen fixture during loading may also cause an uneven stress distribution.

During the tertiary creep stage, primary and secondary slip dislocations alternate, causing distortion of the rafted *γ*′ phases, which leads to a decrease in the strength of both *γ*′/*γ* phases and an increase in the creep rate, ultimately resulting in the macroscopic rupture of the specimen [10]. Using 3D-DIC, which can measure the spatial information of an object, the full-field strain contour diagram along the z-axis was obtained by calculating the specimen image before rupture, as shown in Figure 13. It can be seen that the *t* = 1.2 mm specimen has a larger z-axis strain *ε*_zz_ than the *t* = 0.6 mm specimen, which indicates that a thicker specimen usually has a higher rupture strain and higher damage tolerance.

### 4.3. Fractography

**Figure 14:**
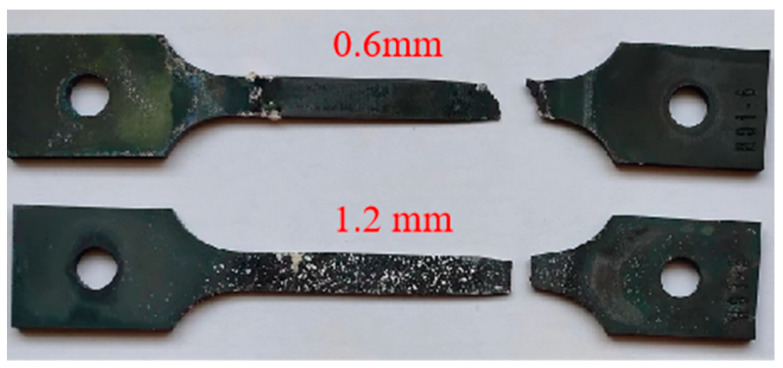
The morphology of the ruptured thin-walled specimens.

The fractography of thin-walled specimens after creep rupture is observed using an optical microscope (OM) and scanning electron microscope (SEM). Figure 14 shows the OM observation of the side fractography of the thin-walled specimens. It can be seen that the *t* = 1.2 mm specimen is mainly affected by Mode-I rupture, while the *t* = 0.6 mm specimen shows a combination of Mode-I rupture and crystallographic rupture. Before rupture, the specimen exhibited obvious necking, and the location of the necking coincided with the final rupture position of the specimen.

**Figure 15:**
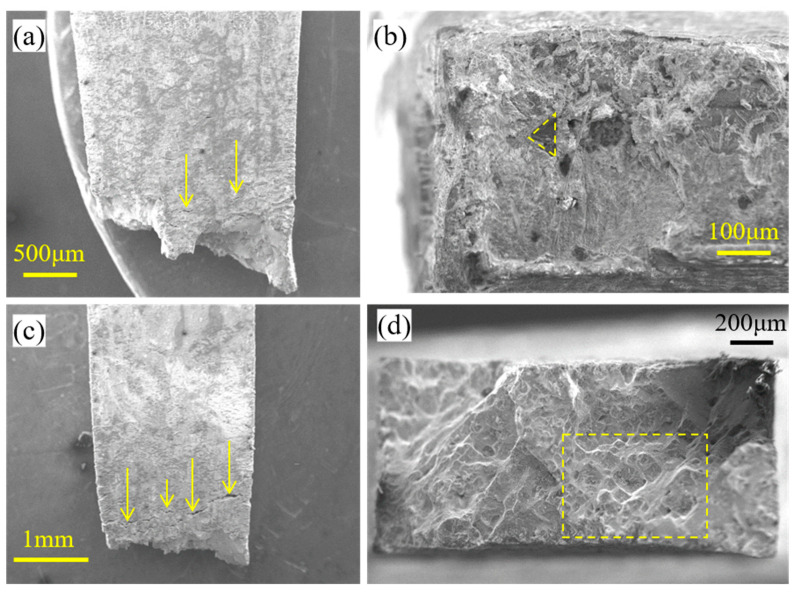
SEM observation of the rupture thin-walled specimens. (**a**) The region near the fracture surface of the 0.6 mm thick specimen reconstructed after rupture. (**b**) The fracture morphology of the left part of one half of the specimen in (**a**) (the loading direction is towards the viewed plane). (**c**) The region near the fracture surface of the 1.2 mm thick specimen reconstructed after rupture. (**d**) The fracture morphology of the left part of one half of the specimen in (**c**).

Figure 15 compares the SEM observations of rupture of the 0.6 mm thick and 1.2 mm thick thin-walled specimens tested at 980 °C/250 MPa. The SEM image of half of the ruptured thin-walled specimen of a 0.6 mm thickness is shown in Figure 15a and that of the 1.2 mm thick specimen is shown in Figure 15c. Surface cracks are observed on the side surface of the specimens as marked by yellow arrows. After creep rupture, both the 0.6 mm thick and 1.2 mm thick thin-walled specimens showed a gradual reduction in the cross-sectional area, with the cross-sectional area near the rupture section being roughly 20~28% less than that of the far gauge section. It is worth nothing that the 1.2 mm thick specimen has a rupture surface nearly parallel to the cross-section and 44.2% elongation, while the 0.6 mm thick specimen has an inclined rupture surface and only 16.4% elongation. These findings suggest that a thinner specimen has more brittleness in high-temperature creep rupture tests.

**Figure 16:**
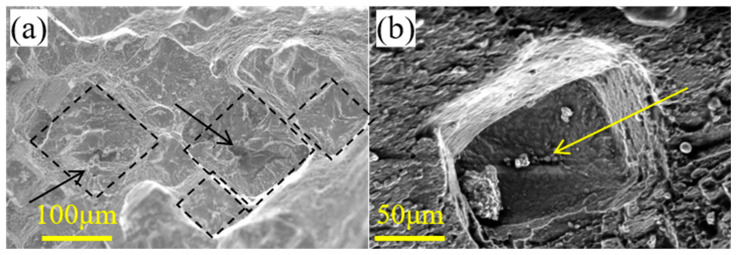
Detailed rupture morphology of the 1.2 mm thick thin-walled specimen. (**a**) The detailed morphology of the left part of one half of the specimen. (**b**) The detailed morphology of the cleavage plane.

The detailed rupture surface of a 0.6 mm specimen is shown in Figure 15b, which shows the presence of deformed internal microvoids and surface oxidation. The surface oxidation cracks account for more than 50% of the length of the through-wall crack. In contrast, the zoomed view of the rupture morphology of a 1.2 mm specimen in the inset of Figure 15d shows the presence of microvoids and microcracks in cup-like depressions. The rupture morphology indicates that rupture occurred due to the nucleation, growth, and coalescence of voids and not because of microcracks emerging in the oxide layer, as these cracks were arrested after further oxidation. Square-like cleavage cracks are found in the more detailed morphology, marked by dotted lines and arrows in Figure 16a. For the microvoids located in the center of the cleavage plane, which are marked by arrows in Figure 16a,b, the cleavage stripes with radial features are found near the round-shaped holes and terminate at the boundary of the square-like cleavage. This finding also indicates that when the specimen thickness increases to a certain length, the fracture mode transitions to complete ductile fracture, with high elongation in high-temperature creep rupture.

**Figure 17:**
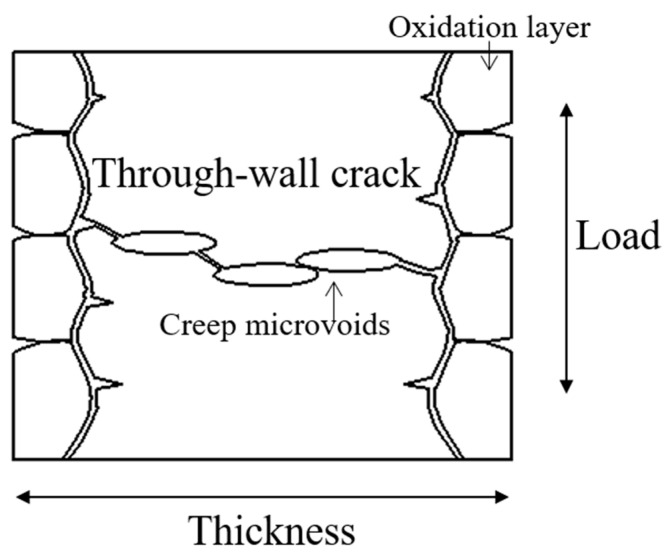
Creep damage mechanism and through-wall crack formation mechanism.

Srivastava [6] divided the creep damage of nickel-based single-crystal thin-walled specimens into surface damage and bulk damage. For creep tests at a high temperature, the surface damage is mainly caused by the loss of an effective bearing area due to surface oxidation, diffusion, and dynamic recrystallization, while the bulk damage is mainly caused by the loss of an effective bearing area due to creep microvoids and microcracks around the void surfaces. Figure 17 presents the damage mechanism and through-wall crack formation mechanism for a thin-walled specimen. For thinner specimens, surface damage plays a greater role, and through-wall cracks are easily formed. According to fracture mechanics, a through-wall crack will lead to fast creep crack propagation and final rupture. Thus, it can be determined that a thinner specimen usually has a lower rupture strain and weaker damage tolerance.

## 5. Conclusions

This study investigated the influence of thickness on the creep performance of a nickel-based single-crystal superalloy, DD6. Creep tests were conducted on specimens with thicknesses of 0.6 mm and 1.2 mm under conditions of 980 °C/250 MPa, and single-camera 3D-DIC was used to measure the full-field deformation. As a result, local strain curves, average creep strain curves, and 3D deformation contour maps of the thin-walled specimens at the rupture points were obtained. The main conclusions of this study are as follows:

(1) The advanced single-camera 3D-DIC system was used to measure translational distance with a relative error of less than 5% in this study. During the creep test under high-temperature conditions of 980 °C, the images collected from 0 to 65 h had correlation coefficients differing by less than 5%, indicating that the high-temperature speckle used in this experiment has good long-term heat resistance. The combination of blue light and a narrowband filter can effectively suppress the influence of thermal radiation on imaging. The filter technique can significantly reduce the impact of thermal flow disturbance on the test results.
(2) Comparing the creep curves of conventional cylinder specimens and thin-walled specimens of different thicknesses, it was found that the creep life of the latter was significantly lower than that of the former, and there was a significant thickness debit effect on specimens of different thicknesses. Basically, before the tertiary creep stage, thinner specimens had higher average creep rates and entered the tertiary creep stage earlier, leading to earlier creep rupture as compared to the thick specimens and cylinder specimens. According to the full-field strain, the creep strain of the edge section of thin-walled specimens is larger than that of the middle section, leading to a lack of coordination in the damage process, which may be a significant cause of the thickness debit effect.
(3) Through the comparison between the local strain curve at the rupture point and the average creep strain curve obtained by DIC method, it was found that during the second creep stage, the local creep rate at the rupture point was less affected by the thickness, while the average creep rate in the working section significantly increased with decreasing thickness, which suggests that the maximum local creep rate is the criterion of the tertiary creep stage. During the tertiary creep stage, both the local and average creep rates at the rupture point and in the working section increased with decreasing thickness. Notably, the increase in the local creep rate was significantly higher than that in the average creep rate. When the specimen ruptured, the rupture strain at the rupture point decreased significantly with decreasing thickness. The deformation contour map also indicated that a thicker specimen usually has a higher average rupture strain and higher damage tolerance. Our comprehensive analysis indicated that the length of the through-wall crack formed due to the thinning of the specimen is the primary factor affecting the damage tolerance performance.

## Figures and Tables

**Table 1:** Material properties of DD6 [001] alloy at 25 °C and 980 °C [23].

	Temperature	25 °C	980 °C
Material Parameters			
Elastic Modulus *E* (GPa)		131.5	80.5
Yield Strength $\sigma_{p0.2}$ (MPa)		965	732
Tensile Strength $\sigma_{b}$ (MPa)		1001	802
Elongation $\delta_{5}$ (%)		16.1	32.6
Reduction of Area *φ* (%)		17.1	41.9

**Table 2:** Accuracy verification results of single-camera 3D-DIC test system.

Displacement Value Measured by a Scale/ (mm)	Displacement Value Measured by DIC/(mm)	Absolute Error/(mm)	Relative Error/(%)	Variance of DIC Measurements/ (mm)
2	1.914	0.086	4.3%	0.00062
4	4.096	0.096	2.4%	0.00022
6	5.932	0.068	1.1%	0.00037
8	7.927	0.073	0.91%	0.00049
10	9.912	0.088	0.88%	0.00056
11	10.914	0.086	0.78%	0.00039

## Data Availability

The data that support the findings of this study are available from the corresponding author, wxu621@163.com (X.W.), upon reasonable request.
